# Tales from the crypt: intestinal niche signals in tissue renewal, plasticity and cancer

**DOI:** 10.1098/rsob.180120

**Published:** 2018-09-12

**Authors:** Maureen Spit, Bon-Kyoung Koo, Madelon M. Maurice

**Affiliations:** 1 Cell Biology, Center for Molecular Medicine, UMC Utrecht, Heidelberglaan 100, 3584 CX Utrecht, The Netherlands; 2 IMBA – Institute of Molecular Biotechnology, Dr Bohr-Gasse 3, 1030 Vienna, Austria; 3 Oncode Institute, The Netherlands

**Keywords:** intestinal stem cells, signalling, microenvironment, colorectal cancer

## Abstract

Rapidly renewing tissues such as the intestinal epithelium critically depend on the activity of small-sized stem cell populations that continuously generate new progeny to replace lost and damaged cells. The complex and tightly regulated process of intestinal homeostasis is governed by a variety of signalling pathways that balance cell proliferation and differentiation. Accumulating evidence suggests that stem cell control and daughter cell fate determination is largely dictated by the microenvironment. Here, we review recent developments in the understanding of intestinal stem cell dynamics, focusing on the roles, mechanisms and interconnectivity of prime signalling pathways that regulate stem cell behaviour in intestinal homeostasis. Furthermore, we discuss how mutational activation of these signalling pathways endows colorectal cancer cells with niche-independent growth advantages during carcinogenesis.

## 1. Adult stem cells are critical for tissue homeostasis

Adult tissue homeostasis strictly depends on the balanced generation of new cells that replenish cells that are lost through natural attrition or tissue injury. This process of tissue regeneration is fuelled by small populations of stem cells that are defined by their unique ability to renew themselves persistently (self-renewal) while also giving rise to the specialized cell types of the pertinent tissue (multipotency) [1–3]. These adult stem cells are generally referred to by their tissue of origin (e.g. haematopoietic, neuronal or intestinal stem cells (ISCs)). Depending on local needs, stem cells may switch their mode of cell division from symmetric to asymmetric. Symmetric division gives rise to two identical daughter cells, both endowed with stem cell properties. Asymmetric division produces only one stem cell and a progenitor cell via signals from the microenvironment and unequal segregation of proteins or RNA, which direct distinct gene expression profiles that control the fate of the newly generated cell [4,5].

Stem cell activity is for a large part dictated externally by the microenvironment (the stem cell niche) to precisely control stem cell output and meet the homeostatic or regenerative demands of the tissue. Extracellular cues, provided by neighbouring niche cells, locally interact with stem cells to regulate their fate by activating specific signalling pathways. Here, we review current knowledge on how stem cells receive and interpret extracellular signals from their niche, focusing on the prototype model of ISCs, which undergo rapid self-renewal kinetics and give rise to the multiple specialized lineages of the intestinal epithelium [6].

## 2. Intestinal architecture

**Figure 1.**
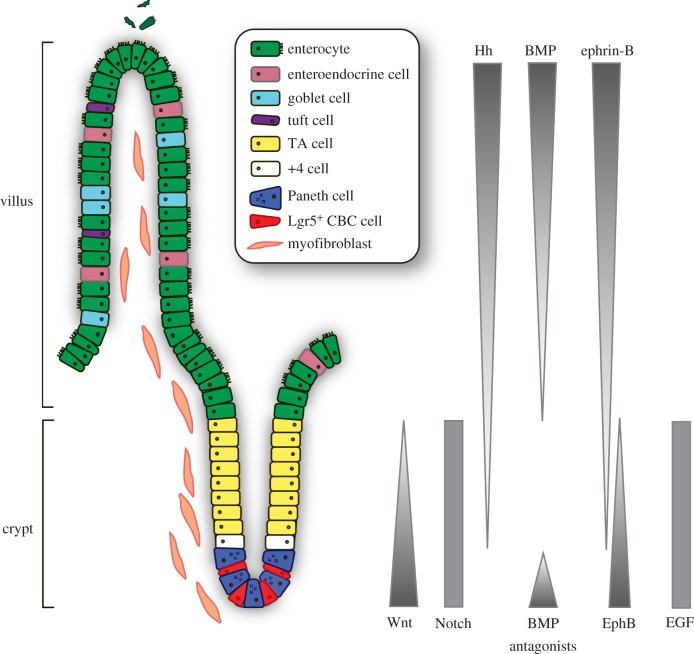
Architecture of the small intestine and the controlling signalling pathways. Actively cycling Lgr5-positive crypt base columnar (CBC) stem cells reside at the bottom of the crypt intermingled with Paneth cells. The stem cells give rise to transit amplifying (TA) cells that terminally differentiate towards all epithelial lineages of the villus. Position 4 (+4) stem cells are mobilized upon tissue damage. Intestinal homeostasis is governed by an interconnected network of signalling pathways, regulating the balance between proliferation and differentiation.

The intestinal mucosa has evolved to absorb water and nutrients while at the same time protecting the body from toxic contents of the gut lumen. The continuous renewal of the gut epithelium allows cells towards the end of their lifetime to shed off at the tip of the villus, while newly produced and differentiated cells migrate up and restock the epithelial barrier. This endless process is sustained by symmetrically dividing stem cells that reside at the crypt base (figure 1).

The intestinal epithelial lining represents one of the most intensively self-replenishing organs; within 5 days the entire epithelial layer is renewed [7]. The architecture of the intestine is designed to maximize the surface for nutrient uptake and is folded into large numbers of villi and crypts in the small intestine. The colon is also folded into crypts but does not display villi. ISCs reside at the bottom of the crypts and are able to replenish the whole crypt–villus axis, generating all differentiated cell types required for the physiological function of the intestine (figure 1). Newly born cells first give rise to the rapidly proliferating subset of progenitors, also known as transit amplifying (TA) cells, that occupy the crypts and expand the population required for epithelial turnover. They migrate upwards while differentiating into one of the specialized epithelial lineages [8]. Among the differentiated cell types, nutrient absorbing enterocytes make up the majority of cells lining the villi. Other major lineages are secretory cell types such as goblet cells that produce mucus to generate a protective barrier and enteroendocrine cells that secrete various hormones that exert both local and systemic regulatory effects. Furthermore, specialized Paneth cells escape the upward flow and migrate downward to constitute the niche for ISCs at the crypt base [9,10], secreting antimicrobial peptides and essential factors for stem cell maintenance. Finally, two more rare cell types are produced, comprising secretory Tuft cells that serve as sensors for luminal contents and initiate type 2 immune responses to helminth infections [11–14], and M (microfold) cells that reside in specialized epithelium overlying Peyer's patches to communicate with the gut's immune system [15]. The continuous proliferation of crypt cells is ultimately balanced by shedding of apoptotic cells at the tip of the villus into the lumen (figure 1).

## 3. Intestinal stem cells

### 3.1. Plasticity of intestinal stem cells

At present, several populations of ISCs have been described based on their markers and localization in the crypt. Among these are the fast-cycling crypt base columnar (CBC) stem cells that are marked by leucine-rich-repeat containing G-protein coupled receptor 5 (Lgr5) [1,7,16]. In addition, a slow dividing, ‘reserve stem cell’ population was identified, also called position 4/+4 cells or label-retaining cells (LRCs) [16–19].

The Lrg5-positive CBC cells that divide every day are considered the driving force of intestinal tissue renewal. Lineage tracing experiments in mice showed that all epithelial cell types originate from the CBC cells that produced clonal ribbons of progeny with lifelong perseverance [1]. To date, Lgr5 has been validated as a bona fide stem cell marker not only in the intestine but also in the stomach pylorus [20] and corpus [21], and hair follicle [22]. Expression profiling of sorted intestinal Lgr5-positive cells provided a CBC stem cell gene expression signature [23,24], which allowed for further functional analysis of additional stem cell genes, such as *Achaete-scute complex homolog 2* (*Ascl2*) [8,25,26], *tumour necrosis factor receptor superfamily member 19* (*TNFRSF19*) or *Troy* [27], *Olfactomedin 4* (*Olfm4*) [28] and *SPARC related modulator calcium binding 2* (*Smoc2*) [23].

The pool of slow cycling reserve stem cells is considered to comprise quiescent stem cells that are mobilized upon tissue damage [18,19]. Several markers for these cells were identified, including *polycomb protein B lymphoma Mo-MLV insertion region 1 homolog (Bmi1)* [29], *telomerase reverse transcriptase (Tert)* [30], *homeobox-only protein (Hopx)* [31] and *leucine-rich repeats and immunoglobulin-like domains 1 (Lrig1)* [32,33].

Additionally, several secretory progenitor populations showed the ability to de-differentiate and revert to stem-like cells to replenish the crypt upon extensive tissue damage. This property was ascribed to LRCs [34] as well as to progenitors that express the Notch ligand Delta-like 1 (Dll1) [35], and to Paneth cells upon irradiation [36]. Furthermore, in addition to cells of the secretory lineage, a recent study showed that the abundant enterocyte progenitors of the absorptive lineage can dedifferentiate and replace lost ISCs upon ablation of Lgr5-expressing stem cells as well [37].

In conclusion, crypt cells display substantial plasticity, employing CBC stem cells for regular tissue renewal and reserve stem cells to act upon tissue damage. Stemness, therefore, appears extrinsically imposed on cells, placing niche signals centre stage for regulating ISC function and intestinal homeostasis.

### 3.2. Lgr5-positive crypt base columnar stem cells

In this review, we refer to Lgr5-positive CBCs when discussing ISCs. Lgr5-positive CBC stem cells divide once a day, generating new CBC cells that reside at the crypt base as stem cells [38]. Owing to the limited space in the crypt base, however, half of the ISCs are randomly pushed out of the niche to become committed progenitor cells, a process called ‘neutral competition’ [38,39]. In this model, all ISCs initially carry the same properties and therefore have a similar chance to persist as an ISC. Real-time intravital imaging confirmed this mechanism *in vivo* [39]. However, detailed quantitative analysis of individual clonal ISC lineages showed that ‘central cells’ at the crypt base have an advantage over ‘border cells’ in the upper rim of the crypt niche for long-term persistence. Border cells were more likely to be displaced into the transit-amplifying compartment, lose their stem cell properties and differentiate along the crypt–villus axis [39]. The spectrum of stem cell activity displays heterogeneity, even within the pool of cells expressing Lgr5. These cells are probably able to transit between states of variable competence, directed by niche-derived signals [39].

## 4. Intestinal stem cell niche

What constitutes and determines the niche for ISCs? The stem cell niche provides a nurturing and guiding environment that sustains the self-renewing, multipotent stem cell population. At the same time, the niche provides local cues for the generation and positioning of differentiated progeny. The ISC niche is constituted by neighbouring Paneth cells within the epithelial layer, and by myofibroblasts, fibroblasts, neuronal and smooth muscle cells within the subepithelial mesenchyme that tightly line the crypt base basal lamina and the extracellular matrix [10,40,41] (figure 1). The close association and direct contact of these niche cells with ISCs facilitates the supply of essential factors for ISC maintenance and proliferation. The subepithelial mesenchyme produces various Wnts and epidermal growth factor (EGF) [42–44]. Furthermore, these cells provide R-spondins, potent Wnt signalling agonists, and Noggin, gremlin 1/2 and chordin-like 1, inhibitors of bone morphogenetic protein (BMP), to repress BMP-mediated differentiation [40,42,45–47]. Recently, subepithelial telocytes were demonstrated to be a vital source of Wnt ligands, as blockage of Wnt secretion from these rare, large cells results in impaired epithelial renewal and disruption of intestinal integrity [48,49]. Similarly, subepithelial Gli1-positive mesenchymal cells provide a crucial source of Wnts, as blockage of Wnt secretion from these cells also results in stem cell loss and subsequent loss of colonic epithelium integrity, which ultimately leads to epithelial death [50]. In addition, within the epithelium, Paneth cells provide essential growth signals, including Wnt3, EGF and Notch ligands, described in detail below [10,42]. Interestingly, ablation of Paneth cells does not result in ISC depletion *in vivo*, but *in vitro* cultured mini-guts (intestinal organoids), however, lack the mesenchymal component and as such fully depend on Wnt3 production by Paneth cells for stem cell maintenance and renewal of the epithelium [10,51]. These combined findings show that both mesenchymal cells, especially telocytes and Gli1+ cells, and Paneth cells serve as important sources for growth factors in the control of tissue renewal.

**Figure 2.**
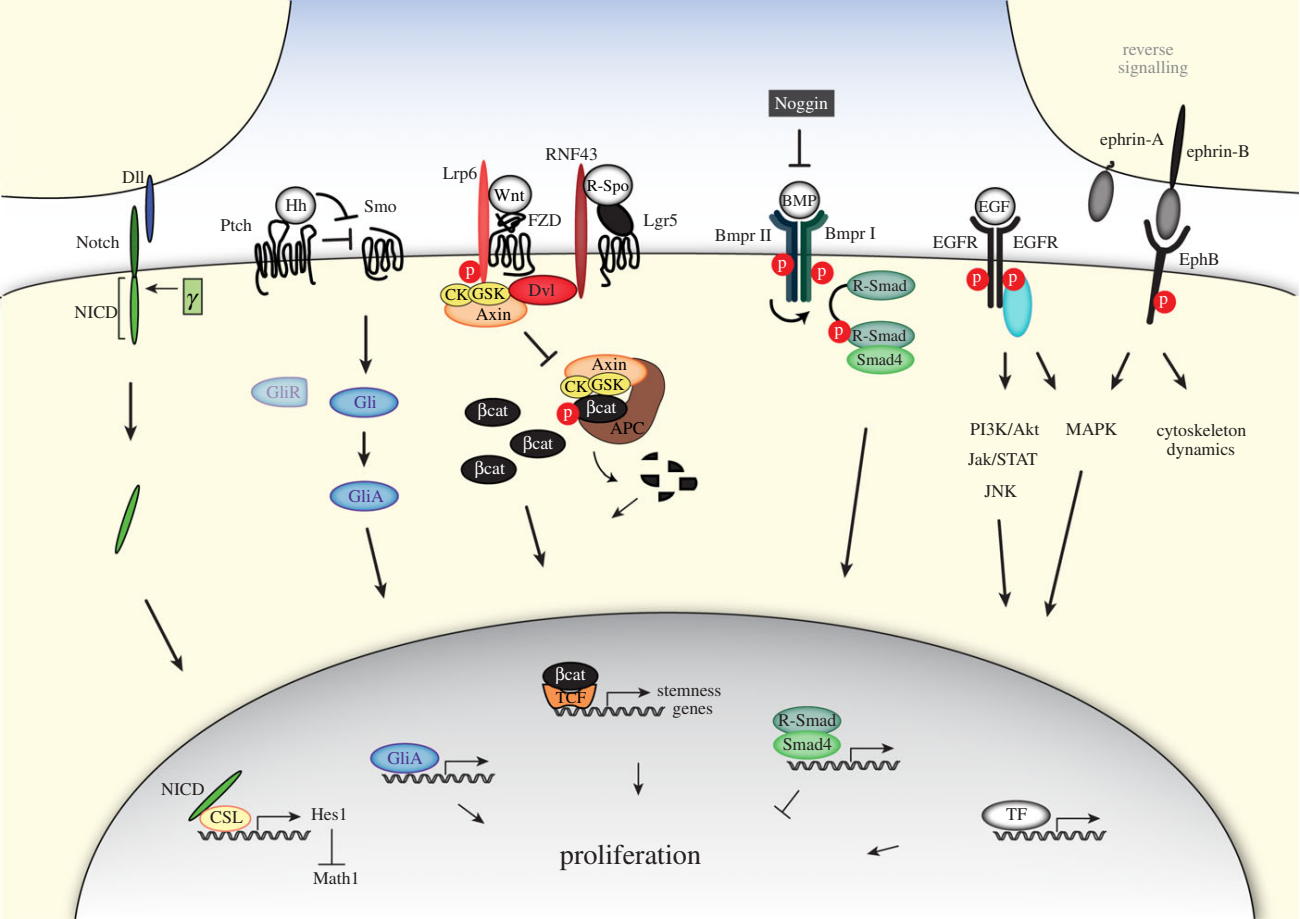
Multiple key signalling pathways govern intestinal homeostasis. Representation of the principal signalling cascades of Notch, Hedgehog (Hh), Wnt, bone morphogenetic protein (BMP), epidermal growth factor (EGF) and Eph–ephrin that together control stem cell behaviour and intestinal homeostasis, see text for further details. Dll, delta-like ligand; NICD, Notch intracellular domain; *γ*, γ-secretase; Hh, Hedgehog; Ptch, Patched; Smo, Smoothend; Gli, glioblastoma; GliR, Gli repressor; GliA, Gli activator; Lrp6, low-densitiy lipoprotein receptor-related protein 6; FZD, Frizzled; RNF43, RING finger protein 43; Lgr5, leucine-rich-repeat containing G-protein coupled receptor 5; R-spo, R-spondin; CK, casein kinase; GSK3, glycogen synthase kinase; Dvl, Dishevelled; p, phospho-group; APC, adenomatous polyposis coli; βcat, β-catenin; TCF, T cell-specific transcription factor; BMP, bone morphogenetic protein; Bmpr I/II, BMP type I or II receptor; EGF, epidermal growth factor; EGFR, EGF receptor; PI3K, phosphoinositide 3-kinase; Jak, Janus kinase; STAT, signal transducer and activator of transcription; JNK, c-Jun N-terminal kinase; MAPK, mitogen-activated protein kinase; CSL, CBF1, suppressor of hairless, Lag-1; Math1, Atoh1, atonal homolog 1; Hes1, hairy and enhancer of split 1; TF, transcription factor.

Thus, ISCs and daughter cells are subjected to and directed by a broad array of signals present in their niche. Polarized gradients of these mesenchymal- and epithelial-derived signals exist both in the crypt and also along the crypt–villus axis (figure 1). The balance between the generation of new cells and their functional specialization is regulated by numerous signalling pathways, which control proper ISC maintenance and intestinal architecture. Among these are the Wnt/β-catenin, Notch, Hedgehog, BMP, EGF and Eph–ephrin signalling cascades (figure 2). Below, we review these pathways and how their interconnected circuitry governs intestinal homeostasis.

## 5. Wnt signalling controls maintenance and size of the intestinal stem cell zone

### 5.1. Wnt signalling

The conserved Wnt signalling pathway determines crucial developmental processes and, importantly, controls tissue homeostasis in adult organisms. It has emerged as a pivotal player in the specification and maintenance of stem cell compartments in a wide array of tissues and organs. In the intestine, Wnt signalling is the main driving force of crypt proliferation.

Wnt ligands, produced by both Paneth and surrounding stromal cells, bind to their cognate receptors Frizzled (FZD) and low-density lipoprotein receptor-related protein 5/6 (Lrp5/6) at the surface of adjacent stem cells. Subsequent activation of the canonical Wnt pathway leads to the accumulation and nuclear entry of the transcriptional co-activator β-catenin to drive the expression of target genes involved in stem cell maintenance [6,52]. Wnt-induced stabilization of β-catenin involves the inactivation of a large multi-protein complex composed of the scaffold proteins Axin and adenomatous polyposis coli (APC) as well as the kinases glycogen synthase kinase 3β (GSK3β) and casein kinase 1 (CK1). In unstimulated cells, this destruction complex captures β-catenin and earmarks it for proteolysis through Ser/Thr phosphorylation of its flexible N-terminus [53,54]. Recognition of phospho-β-catenin by the ubiquitin ligase β-TrCP subsequently mediates its rapid ubiquitin-mediated proteasomal degradation [55,56].

Binding of Wnt to the FZD and Lrp6 receptors at the cell surface interferes with β-catenin degradation by a number of molecular rearrangements. After formation of a trimeric Wnt–FZD–Lrp5/6 complex, the cytoplasmic effector protein Dishevelled (Dvl) is recruited. Next, the activated receptor complex captures the destruction complex organiser Axin, probably through heterodimerization of the Axin and Dvl DIX domains [57–59]. The Axin-associated kinases GSK3β and CK1 turn their activity to the cytoplasmic tail of Lrp6, which, upon phosphorylation, provides a docking site for further Axin proteins. The redistribution of Axin–kinase complexes to the plasma membrane is considered a key step in the inactivation of β-catenin destruction. As a consequence, the pool of intracellular β-catenin increases and migrates to the nucleus to bind the T-cell factor (TCF) family of DNA-bound transcription factors and induces transcription of Wnt target genes (figure 2) [60]. Among the earliest target genes discovered is *c-Myc*, a well-known driver of proliferation of undifferentiated cells [61]. The Wnt target gene list has vastly expanded ever since, revealing multiple layers of positive and negative feedback regulation in the control of stem cell identity [23,62–65].

### 5.2. Wnt signalling in the intestine

The first clues for the crucial role of Wnt in the intestine originated from mouse genetic experiments. Neonatal mice deleted for *TCF4*, one of the main downstream effectors of Wnt, completely lack proliferative crypts, illustrating the requirement of Wnt signalling for establishment and maintenance of the stem cell compartment [66]. Maintenance of adult crypt proliferation remains dependent on Wnt signalling as conditional deletion of TCF4 in adult mice resulted in the loss of nearly all proliferating crypts, coinciding with progressive loss of Wnt target gene expression [67]. Furthermore, conditional deletion of β-catenin as well as overexpression of the diffusible Wnt inhibitor Dickkopf 1 (Dkk1) results in complete ablation of intestinal crypts in the adult mouse [68–71]. Moreover, transgenic expression of R-spondin 1 (R-Spo1), a strong Wnt agonist that acts through the Lgr4/5–Wnt receptor complex (described in more detail below), results in a massive hyperproliferation of intestinal crypts [72]. On the other hand, simultaneous deletion of both Lgr4 and Lgr5, the receptors for R-Spo, leads to the disappearance of crypts [73].

The fact that Wnt signalling plays a vital role in ISC maintenance is further illustrated by the nuclear β-catenin levels that are highest at the crypt base and gradually decrease along the crypt–villus axis [74,75]. Concordantly, the expression of various Wnt ligands (Wnt3, Wnt6 and Wnt9b) as well as their cognate receptors FZD5/7 is also highest at the crypt base [42,43,76,77]. Wnts produced by Paneth cells decorate the membranes of adjacent stem cells by binding to the highly expressed FZD receptors [78]. Through the regulation of FZD turnover and cell division, the membrane-bound reservoir of Wnts at the crypt bottom is gradually diluted, sculpting a gradient of Wnt along the crypt–villus axis [78]. Accordingly, Wnt target genes display maximum expression in the crypt base and gradually decrease moving upward along the crypt domain [40]. Interestingly, Paneth cells themselves also depend on Wnt signals [42,77,79] and require expression of the Wnt target gene *Sox9* for their development and formation [80,81].

### 5.3. Stem cell-specific Wnt target genes

As described above, ISCs are marked by the Wnt target gene *Lgr5*. Transcriptome and proteome analysis of sorted Lrg5-positive cells unveiled multiple Wnt target genes among the stem cell-specific gene set [23,82]. Many of these genes have proven to be essential for stem cell maintenance and activity, regulating both positive and negative feedback signalling loops.

Among the ISC-specific Wnt target genes are the transmembrane receptor tyrosine kinase genes *EphB2* and *EphB3* [63]. Their expression is highly enriched on ISCs and Paneth cells, while transcription of their repulsive ligand, ephrin-B1, is concomitantly repressed in the crypts. Only upon exiting the crypt do cells start to express ephrin-B1 as a result of the decline of Wnt signals. Subsequent repulsive interactions between EphB-positive and ephrin-B-positive cells results in segregation of these cells, thereby controlling correct positioning of the cells. Indeed, EphB2/3 deficiency in mice results in a random localization of cells, including Paneth cells, along the crypt–villus axis. Hence, Wnt signalling controls architectural integrity of the stem cell zone [63,83–85].

The Wnt target gene *Ascl2* is expressed in a Wnt-dependent and highly restricted fashion in ISCs [23–25,62,82,86]. Conditional knockout of *Ascl2* in the adult intestinal epithelium leads to the elimination of CBC stem cells, whereas ectopic intestinal expression of Ascl2 induces hyperproliferation of crypts and de novo cryptogenesis in villi [62]. For this reason, Ascl2 is suggested to be a master regulator of the crypt stemness programme. Recent research showed that Ascl2 cooperates with β-catenin/TCF to activate the genes fundamental to the stem cell state. Ascl2 is activated when cells reach a specific Wnt/R-Spo signalling threshold and, as Ascl2 is capable of self-activation, is suggested to translate the Wnt gradient present in the crypt into a discrete ‘on’ or ‘off’ decision for stemness [25].

Another example of a stem cell-specific Wnt target gene is *Tnfrsf19* or *Troy,* which is proposed to interact with Lgr5 and to negatively regulate Wnt/R-Spo signalling. As such, Troy is proposed to constitute a negative feedback loop to avoid over-activation of Wnt signalling, thereby preventing subsequent crypt enlargement and ultimately tumourigenesis [27].

In addition, the stem cell-specific and homologous Wnt target genes *Ring finger 43* (*RNF43*) and *zinc and ring finger 3* (*ZNRF3*) were shown to act in a negative feedback manner in the gut [87,88], as discussed below.

In summary, Wnt signalling constitutes the major driving force behind homeostatic self-renewal of the crypt through regulation of expression of critical regulatory genes.

### 5.4. Wnt pathway regulation by R-spondin and RNF43/ZNRF3

R-spondins are a group of small, secreted proteins (R-Spo1–4) that strongly potentiate Wnt/β-catenin signalling [72,89–93]. R-Spo proteins function as stem cell growth factors and can promote tissue regeneration [72,94]. Despite their biological significance there is no consensus on the exact mechanism by which R-Spo increases Wnt signalling. Various membrane proteins were proposed to act as R-Spo receptors, including Wnt receptors FZD and Lrp6 [95,96], Kremen [97], syndecan 4 [98], Lgr4/5 [73,99,100] and membrane E3 ubiquitin ligases RNF43/ZNRF3 [88,101–107].

To date, Lgrs are largely accepted as the primary receptors for R-Spo. Lgrs belong to the seven-span transmembrane receptor family and are known for their large extracellular leucine-rich repeats domain that is involved in ligand binding [108]. All four members of the R-Spo family can bind to the leucine-rich domain of Lgr4, Lgr5 and Lgr6 [73,99]. Lgr proteins have been identified as components of the Wnt receptor complex, and R-Spo binding to Lgr is hypothesized to stimulate the formation of higher order receptor complexes, thereby leading to enhanced Wnt signal transduction [73].

A new hypothesis emerged with the discovery of the two homologous genes *RNF43* and *ZNRF3* that display strongly enriched expression in the ISC population [87,88]. These genes encode for single-span transmembrane E3 ubiquitin ligases and operate as potent negative feedback regulators of Wnt signalling to control aberrant expansion of the crypts. Upon genetic removal of these negative regulators of Wnt signalling in the intestinal epithelium, mice showed very rapid tumour formation in the intestine. RNF43 and ZNRF3 inhibit Wnt signalling by targeting the Wnt receptors FZD and Lrp6 at the cell surface for ubiquitin-mediated endocytosis and lysosomal degradation, regulating cellular sensitivity for incoming Wnt ligands [87,88]. The underlying mechanism of RNF43/ZNRF3-mediated Wnt receptor targeting remains unknown but involves the cytoplasmic effector Dvl that was found to interact with the cytoplasmic tail of RNF43 [109]. Another regulatory layer emerged through the discovery that both RNF43 and ZNRF3 interact with R-Spo [101,104,106,110–114]. In current models, binding of R-Spo to Lgr4 recruits the RNF43/ZNRF3 receptors and induces their membrane clearance [88,105]. R-Spo-mediated removal of RNF43/ZNRF3 leads to stabilization of Wnt receptors at the cell surface, strongly enhancing cellular responses to Wnt (figure 2) [105].

A recent study demonstrates a distinct, non-redundant cooperation between Wnt and R-Spo ligands in ISC homeostasis and suggests that Wnt ligands act as priming factors that confer basal proliferative competence to ISCs by maintaining R-Spo receptor expression, which then drives the further expansion of stem cells via R-Spo ligands present in the niche [115].

In summary, the importance of Wnt signalling in collaboration with R-Spo as a major driving force of crypt proliferation is underscored by its tight regulation through multiple positive and negative regulatory feedback loops. Both R-Spo and RNF43/ZNRF3 represent prime examples of Wnt regulators with key functions in intestinal homeostasis.

## 6. Notch signalling regulates cell fate decisions and stemness in the crypt

The Notch signalling cascade is a highly conserved cell communication pathway that directs cell fate decisions in multicellular organisms. The mammalian Notch family comprises four single-span transmembrane Notch receptors (Notch1–4) and five single-span transmembrane Delta/Serrate/Lag2 (DSL) ligands (Jagged (Jag) 1 and 2, Delta-like (Dll) 1, 3 and 4). Notch signalling is triggered via direct cell-to-cell contact, through which the membrane-bound ligands exposed at the juxtaposed cell membrane bind and activate the Notch receptor. This ligand–receptor engagement results in the initiation of several proteolytic steps that modulate Notch receptor activity. Ultimately, the Notch intracellular domain (NICD) is released through γ-secretase protease activity (reviewed in [116,117]). Subsequently, NICD translocates to the nucleus to drive gene expression upon binding of the transcription factor CSL (CBF1, suppressor of hairless, Lag-1). CSL represses transcription of target genes in unstimulated cells but is converted into a transcriptional activator upon binding of NICD (figure 2) [118].

In the intestinal crypt, Notch signalling critically regulates the cell fate decision between absorptive and secretory cell types. This is illustrated by the use of γ-secretase inhibitors, which block Notch receptor signalling and mediate a massive conversion of proliferative cells into secretory cells [119,120]. Conversely, enhancement of Notch signalling via intestine-specific transgenic expression of NICD blocks the commitment of cells to adopt a secretory lineage fate [121].

The switch between lineages is mainly decided by two basic helix-loop-helix transcription factors. Notch signalling induces expression of the transcription factor Hes1 (hairy and enhancer of split 1) that in turn antagonizes the transcription factor Math1 (or Atoh1 (atonal homolog 1)) [122]. Math1 is the crucial regulator of the transcriptional programme for secretory lineage differentiation. Its depletion results in a complete absence of goblet, Paneth and enteroendocrine cells in the intestine [123–125], and Math1 overexpression directs progenitor cells into the secretory lineage [126]. Math1 expression is restricted to the secretory cells of the intestine, while Hes1 is expressed in most proliferative crypt cells. Inhibition of Notch signalling rapidly decreases Hes1 expression and results in upregulated Math1 expression in all crypt cells [120]. In line, deletion of Hes1 is associated with an excess of secretory cells at the expense of enterocytes [122]. Notably, similar phenotypes are observed upon simultaneous deletion of *Notch1* and *Notch2* genes and genetic ablation of CSL [120,127]. In summary, Notch signalling induces Hes1 levels to suppress Math1-dependent differentiation towards secretory lineages (figure 2).

How does Notch signalling affect ISCs? Lgr5-positive CBC cells predominantly express Notch1 and Notch2 receptors at the cell surface and Notch1 receptor mRNA was found enriched in these cells, signifying a regulatory role of Notch within the CBC stem cell compartment [62,128]. Neighbouring Paneth cells express Notch ligands Dll1 and Dll4, providing the ligands to activate Notch signalling in ISCs [10,129]. Notably, lineage-tracing experiments of cells undergoing active Notch signalling identified long-lived progenitors able to give rise to all the mature epithelial cell types [129,130]. Inhibition of Notch signalling results in rapid loss of CBC cells, indicating its requirement for stem cell proliferation and survival [131]. In particular, the stem cell-specific marker *Olfm4* was shown to be a direct target gene of Notch signalling [131]. Thus, active Notch signalling is crucial for maintenance and activity of the ISC pool. Recently, it was shown that Notch signalling also plays a crucial role in intestinal renewal upon injury by irradiation. Interestingly, Paneth cells were shown to dedifferentiate and proliferate and start to line the crypt–villus axis. This newly gained stem cell-like capacity of Paneth cells is attributed to activated Notch signalling in the Paneth cells themselves [36].

In summary, Notch signalling regulates different aspects of intestinal homeostasis, stimulating both stem cell maintenance and cell fate determination of progenitor cells, and can induce Paneth cells to dedifferentiate upon tissue damage.

## 7. Hedgehog signalling regulates intestinal mesenchyme

The Hedgehog (Hh) family of secreted ligands consists of three subgroups; the Desert Hedgehog (Dhh), Indian Hedgehog (Ihh), and Sonic Hedgehog (Shh) groups [132]. Binding of Hh to the twelve-pass transmembrane receptors Patched 1 or 2 (Ptch1/2 or Ptc1–2) activates a signalling cascade that ultimately drives the activation of the zinc-finger transcription factor glioblastoma (Gli) (Gli1, Gli2 and Gli3), leading to the expression of Hh specific target genes. In the absence of Hh ligands, Ptch blocks the activity of the seven-span transmembrane protein Smoothened (Smo), and full-length Gli proteins are proteolytically processed to C-terminally truncated ‘GliR’ (Gli repressor) that actively repress a subset of Hh target genes. Binding of Hh to Ptch results in loss of Ptch activity and subsequent activation of Smo, which transduces the Hh signal to the cytoplasm, blocks the production of GliR and induces the conversion of Gli proteins into transcriptional activators (GliA) and thereby induces target gene transcription (figure 2) [132–136]. Target genes include *Ptch1/2*, *Gli1*, *Hedgehog-interacting protein (Hhip)* for feedback regulation [132–134,137,138], and genes that drive proliferation and survival, such as *cyclin D1*, *myc* and *Bcl2* [135,139,140].

In the intestine, Ihh is the main hedgehog expressed. Low levels of Shh may be expressed at the base of the small intestinal and colonic crypts [141–144]. Ihh is secreted in a paracrine manner by epithelial cells to act on the surrounding mesenchymal cells, including smooth muscle precursor and myofibroblast cells [142,145]. In addition, intestinal macrophages and dendritic cells may directly respond to Hedgehog signalling [145]. Ihh and Shh ligands secreted by TA cells interact with Ptch receptors localized on mesenchymal cells to induce BMP production [144,146,147]. BMPs negatively regulate intestinal epithelial proliferation and restrict the number of stem cells in the crypt [40,148,149] (see paragraph below). Furthermore, (Hh downstream factor) Gli1-positive mesenchymal cells secrete Wnt ligands that are essential for stem cell renewal in the colon and in the small intestine can act as a reserve Wnt source [50].

Constitutive activation of Hh signalling, either by systemic deletion of Ptch [144] or by selective overexpression of Ihh in the intestinal epithelium (*Villin-Ihh* transgenic mice) [150], results in an accumulation of mesenchymal cells. Moreover, experiments in which Hh signalling was conditionally lost in the adult intestine showed that Hh not only signals for expansion of the mesenchyme but is also required to maintain smooth muscle and myofibroblast cells [150,151]. Furthermore, analysis of Ihh mutant mice showed that loss of Ihh signalling ultimately results in the loss of smooth muscle precursor cells, leading to complete loss of the villus core support structure [141]. Decreased Hh signalling in the adult intestine was also shown to enhance Wnt pathway activity, thereby compromising differentiation and driving crypt hyperplasia [143].

Thus, in current models, Hh signalling indirectly affects ISCs via (i) induction of repressive BMP signalling and (ii) modulation of adjacent stroma for supportive structure.

## 8. Bone morphogenetic protein signalling regulates crypt formation and terminal differentiation

BMPs were initially discovered for their ability to induce bone formation [152] but are now known to play crucial roles during organ development and tissue homeostasis [153]. BMPs comprise a class of extracellular signalling molecules that belong to the transforming growth factor-β (TGF-β) superfamily of proteins. BMPs signal via the canonical Smad-dependent pathway and can induce various non-canonical signalling pathways as well. In the canonical pathway, BMPs initiate signal transduction by binding to BMP type I and type II receptors (Bmpr1–2) that form a heterotetrameric complex. BMP receptors are single transmembrane proteins that carry serine/threonine kinase activity in their intracellular domains. Upon BMP binding, the constitutively active Bmpr2 transphosphorylates Bmpr1. Subsequently, Bmpr1 phosphorylates the receptor-bound R-Smads1/5/8 (receptor-regulated Smads). Phosphorylated Smad1/5/8 then associate with the core mediator Smad4, and the resulting Smad complex translocates to the nucleus to associate with coactivators or corepressors and regulate gene expression patterns (figure 2) [153,154]. BMP target genes include Msx homeobox genes and the proto-oncogene JunB [155,156]. Pathway activity is regulated by extracellular antagonists, such as Noggin, follistatin or gremlin, that sequester BMP ligands, thereby blocking interaction with BMP receptors [157].

In the intestine, the BMP pathway acts as a negative regulator of crypt formation and drives the terminal differentiation of mature intestinal cells [148,158,159]. BMP-2 and -4 ligands are expressed by both mesenchyme and epithelial villus cells and mainly act on the epithelial compartments that contain differentiated cells expressing BMP receptors [148,160]. Mesenchyme-to-epithelium BMP signalling promotes differentiation of progenitor cells while restraining cell proliferation [149]. BMP signalling within the crypt base stem cell niche is carefully regulated by BMP antagonists, such as gremlin1/2, chordin, Noggin and ANGPTL2, expressed by the mesenchyme surrounding the crypt [40,161,162]. Inhibition of BMP signalling in the mouse villus using transgenic expression of the BMP inhibitor Noggin, results in ectopic crypt formation [148]. Similarly, conditional deletion of Bmp receptor 1A results in hyperproliferative crypts [149]. BMP represses Wnt signalling and is expressed in an opposing gradient along the crypt–villus axis, with highest BMP signalling in the cells at the luminal surface (figure 1) [149].

## 9. EGF signalling is required for intestinal stem cell proliferation

EGF is an extracellular ligand that stimulates cell growth, proliferation, and differentiation by binding to its cognate receptor the epidermal growth factor receptor (EGFR). EGFR is also known as ErbB1/HER1, and is a member of the ErbB family of receptor tyrosine kinases. Upon activation by EGF binding, EGFR undergoes a transition from an inactive monomeric form to an active homodimer. EGFR dimerization stimulates its intrinsic intracellular protein-tyrosine kinase activity. As a result, several tyrosine residues in the C-terminal domain of EGFR are autophosphorylated, which drives downstream pathway activation. Downstream signalling effectors associate with phosphorylated tyrosines in the EGF receptor via their SH2 domains and initiate major cellular pro-survival and proliferation signalling cascades, including the mitogen-activated protein kinase (MAPK), phosphatidylinositol 3-kinase (PI3K)/Akt, c-Jun N-terminal kinases (JNK), Jak/STAT and phospholipase C (PLC) pathways (figure 2) [163,164]. Of note, the small GTPase KRAS (Kirsten rat sarcoma viral oncogene homolog) acts as a crucial central relay in a number of these signalling cascades and is commonly mutated in colorectal cancers [165,166].

EGF signalling is required for proliferation and maintenance of ISCs and is produced in the niche by the surrounding Paneth cells and subepithelial mesenchyme [10,42]. In turn, EGFR is highly expressed in ISCs and in TA cells [167]. Indeed, luminally applied EGF strongly induces proliferation of the small intestinal epithelium in rats [168,169]. By contrast, blockade of EGF signalling in organoids converts actively dividing ISCs into quiescent Lgr5+ reserve stem cells that retain expression of various Wnt target genes [170]. Evidently, tight control is necessary to balance EGF-induced proliferation of ISCs. To this end, ISCs express high levels of the EGFR/ErbB inhibitor leucine-rich repeats and immunoglobulin-like domains protein 1 (Lrig1) transmembrane proteins. Lrig1 is an inducible negative feedback regulator of the ErbB receptor family that mediates the ubiquitination and subsequent degradation of canonical EGFRs [32,33,171]. Accordingly, genetic ablation of Lrig1 in mice results in enhanced EGFR/ErbB expression leading to an increase in ISC numbers and significant expansion of crypts [32,33]. This activity of EGFR signalling in intestinal maintenance is highly conserved, as shown by studies using *Drosophila* [172,173].

In summary, controlled expression of Lrig1 forms a negative feedback loop that allows stem cells to fine-tune their cellular response to EGF ligand-mediated signalling and ensures proper crypt size and tissue homeostasis.

Of note, a recent study demonstrated redundancy between EGF and hepatocyte growth factor (HGF) in the intestine. HGF regulates intestinal homeostasis and regeneration by engaging its receptor MET, and interestingly HGF/MET signalling can fully substitute EGFR signals in intestinal organoid cultures [174].

## 10. Eph–ephrin signalling directs appropriate cell positioning along the crypt–villus axis

Eph–ephrin signalling occurs via direct cell–cell contact and is involved in a wide spectrum of biological processes, including the regulation of cell positioning. Ephrin ligands are divided into two subclasses based on their structure and mode of linkage to the cell membrane (ephrin-A and ephrin-B). Ephrin-A proteins are anchored to the membrane by a glycosylphosphatidylinositol (GPI) linkage and lack a cytoplasmic domain, while ephrin-B proteins pass the membrane by their single transmembrane domain and contain a short cytoplasmic part. Eph receptors constitute the largest family of tyrosine kinase receptors and can be divided into the subclasses EphA and EphB, based on sequence similarity and binding affinities for either ephrin-A or -B [175,176].

A unique feature of Eph–ephrin signalling is that both receptor and ligand are competent to transduce signalling upon interaction. This concept of bidirectional signalling has emerged as an important mechanism by which Ephs and ephrins control cell–cell communication. Eph- and ephrin-mediated signalling are generally referred to as forward and reverse signalling, respectively [176]. Essentially, Eph signalling controls cell morphology, adhesion and migration by modifying the organization of the actin cytoskeleton and influencing the activities of integrins and intercellular adhesion molecules [85,176]. Upon binding of an ephrin ligand to the extracellular domain of an Eph receptor, intracellular tyrosine and serine residues of the receptor are auto-phosphorylated, allowing the cytoplasmic tyrosine kinase to subsequently activate and modulate downstream signalling cascades, such as MAPK, Ras and ERK signalling (figure 2) [85,177].

Although Eph–ephrins were initially studied mainly in a developmental context, their physiological functions in adult tissues are rapidly coming to light. In the intestine, high levels of Wnt signalling induce expression of EphB2 and EphB3 in the lower parts of the crypts with simultaneous transcriptional repression of their repulsive ephrin-B1 ligand [63]. Of note, Notch signalling activates the expression of ephrin-B1 in differentiated intestinal cells [178]. ISCs display high levels of EphB2 expression, while Paneth cells are EphB2 negative but express EphB3 [63,179]. The decline in Wnt cues along the crypt–villus axis results in de-repression of the repulsive ephrin-B1 ligand. At the same time, EphB2 expression progressively declines in TA cells as they migrate upwards. The gradient of repulsive EphB2/3–ephrin-B1 interaction coordinates appropriate cell positioning along the crypt–villus axis with differentiated cells moving upwards towards the villus tip [63,83,180,181]. Differentiated Paneth cells that highly express EphB3 escape this upward flow and move towards the crypt bottom. Indeed, in EphB3 null mice, Paneth cells are not restricted to crypts but spread randomly along the villi in the small intestine [63].

## 11. Interconnectivity of signalling pathways governs crypt–villus homeostasis

Each of the above-described signalling pathways controls intestinal homeostasis, through either a direct or indirect modulation of ISC behaviour. ISCs interpret these signals derived from the niche to ensure the balance between cell loss and cell replenishment, thereby safeguarding tissue maintenance. The interconnectivity of the signalling pathways further secures an appropriate strength and timing of signals within the stem cell niche. The Wnt pathway is the main force behind intestinal epithelium homeostasis and requires tight regulation to prevent hyperproliferation of ISCs. The expression of stem cell regulatory Wnt ligands displays a diminishing slope along the crypt–villus axis. At the +4 position, a local production of Wnt antagonists is observed, including Wnt binding secreted Frizzled-related proteins, likely to keep the reserve stem cells at that position in the quiescent state [76]. In the crypt, the Wnt pathway synergizes with Notch signalling to sustain undifferentiated and proliferative stem and progenitor cells in the crypt. Additionally, both pathways are essential for specific lineage commitment of progenitor cells along the absorptive (Notch) and secretory (Wnt) cellular differentiated states. Interestingly, inhibition of Notch signalling, using Notch blocking antibodies, caused conversion of Lgr5-expressing ISCs to secretory cells, leading to stem cell depletion. This coincided with Wnt pathway upregulation and increased secretory cell differentiation. Repression of canonical Wnt signalling rescued this phenotype, suggesting opposing and interconnected activities of Notch and Wnt signalling to guide gut homeostasis [182].

Furthermore, Wnt signalling induces high crypt-specific expression of EphB2 and EphB3, while the decreasing Wnt gradient along the crypt–villus axis generates an opposing gradient of the repulsive ephrin-B ligand, securing spatial segregation and accurate positioning of distinct cellular compartments within the crypt. In the upper part of the crypt and within the villus, the output of Wnt signalling is modulated by cooperative activity of the paracrine Hedgehog and BMP signalling cascades. As the progenitor cells move upwards from the crypt base, the Hedgehog-induced, mesenchyme-to-epithelium BMP signalling promotes differentiation while restraining cell proliferation. Importantly, at the crypt base, the pro-differentiation activity of the BMP pathway is counteracted by locally secreted mesenchyme-derived BMP antagonists. Also, Paneth cell-derived EGF-induced mitotic signalling at the crypt base is balanced by expression of EGFR inhibitor Lrig1.

**Figure 3.**
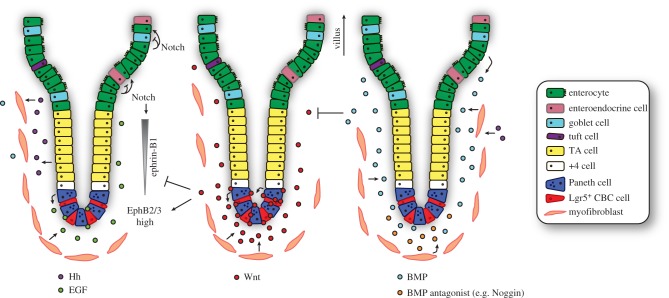
Interconnectivity of signalling pathways governs crypt–villus homeostasis. Intestinal homeostasis and cell fate determination are maintained by interconnectivity of key signalling pathways between epithelial and mesenchymal cells. Wnt ligands (centre) secreted from the Paneth cells and intestinal subepithelial myofibrobasts act predominantly at the base of the crypt to maintain stem cell function and TA cell proliferation. High levels of Wnt signalling induce the expression of EphB2 (ISCs) and EphB3 (Paneth cells), and concurrently repress transcription of the repulsive ligand ephrin-B1. The decline in Wnt signals along the axis results in increased ephrin-B1 levels and proper positioning of the cells. EGF signalling (left) is required for proliferation and maintenance of ISCs and is produced in the ISC niche by the surrounding Paneth cells and subepithelial mesenchyme. Notch signalling regulates cell fate through cell-to-cell contact in the crypt (here for simplicity only visualized on the top left). Notch signalling controls the binary cell fate decision between the secretory and absorptive lineages. Hedgehog (Hh), expressed by epithelial cells in the upper part of the crypt, acts upon and maintains the myofibroblasts. This has a secondary effect on the epithelium through promotion of BMP ligand expression. BMP ligands are predominantly produced by the mesenchymal cells and partly by epithelial villus cells. Mesenchyme-to-epithelium BMP signalling promotes differentiation of progenitor cells while restraining cell proliferation. BMP signalling within the crypt stem cell niche is therefore carefully regulated by BMP antagonists expressed by the mesenchyme surrounding the crypt. Finally, BMP represses Wnt signalling and is expressed in an opposing gradient along the crypt–villus axis.

Collectively, an interconnected network of developmental signalling pathways governs intestinal homeostasis by balancing the processes of cell proliferation and differentiation (figure 3).

## 12. Sequential mutation of ‘niche-like’ signalling pathways drives intestinal carcinogenesis

Disruption of the delicate balance between proliferation and differentiation governed by key signalling pathways in the crypt can lead to hyperproliferation and ultimately tumour growth. Indeed, cancer cells inappropriately turn on a gene programme for self-renewal and survival by acquiring mutations in key components of signalling pathways that are normally provided by the external cues from the niche [183].

The majority of colorectal tumours evolve from benign to malignant lesions by acquiring a series of mutations over time: the adenoma–carcinoma sequence [184,185]. Formation of benign adenomas is initiated by activation of the Wnt signalling pathway, most commonly through inactivating mutations in *APC* [186]. Subsequent activating mutations in the EGFR pathway (*KRAS*, *PIK3CA*), and inactivating mutations in the TGF-β/BMP pathway (*SMAD4*) as well as in p53 (*TP53*) promote progression to an invasive and metastatic phenotype [184,185,187]. Mutations in these genes are presumed to drive colorectal carcinogenesis as they provide selective growth advantages to the mutated cells and are therefore called ‘driver’ mutations. Each human colorectal carcinoma (CRC) is regarded to harbour three to six recurrent driver mutations [187]. Recently, two elegant studies showed, using CRISPR–Cas9-engineered organoid cultures, that a combination of at least four of such driver mutations liberates the ISC from the need of niche factors, rendering gut organoid growth entirely self-sufficient [188,189]. Importantly, such mutant organoids form tumours after transplantation in mice [188,189]. Hence, the introduction of driver mutations in organoids fully copies the CRC phenotype and provides final proof that the sequential disruption of key signalling pathways governs the adenoma–carcinoma sequence.

Activation of the Wnt and the EGFR signalling pathways represent key steps in the initiation and early progression of CRC, by favouring stemness and proliferative characteristics. Subsequent blockade of BMP/TGFβ signalling suppresses differentiation and further tilts the balance towards proliferation of the cancer cells. Ultimately, inactivation of p53 results in loss of DNA damage control and, more importantly, allows CRC cells to escape apoptosis. Together, this establishes an optimal combination of events for cancer cell survival and carcinogenesis.

Interestingly, the combined inactivation of both APC and p53 appears already sufficient to induce extensive aneuploidy, a hallmark of tumour progression. Remarkably, the number of driver mutations in CRC is variable, with a fraction of CRCs carrying only a single pathway alteration [190]. In these cases, acquired microsatellite and/or chromosomal instability and epigenetic changes might alter driver pathway signalling instead.

Clearly, the sequential accumulation of driver mutations drives tumour progression towards CRC, but are the initial genetic alterations also required to maintain CRC cells at later stages of tumour progression? This question was recently addressed by Dow *et al*. [191] using a conditional short hairpin RNA approach to control APC levels in a subset of ISCs in the mouse. The strong reduction of APC levels initiated tumour formation along the intestine, including the colon. Upon restoration of APC expression, tumours regressed and CRC cells underwent differentiation toward normal intestinal cell types, thereby reinstating crypt–villus homeostasis. Strikingly, invasive tumours harbouring additional *KRAS* and *TP53* mutations were also reverted to normal functioning cells after reintroduction of APC [191]. This illustrates that loss of APC is not only essential for CRC onset but remains critical for CRC maintenance even in the presence of sequential driver mutations.

## 13. Concluding remarks

In this review, we summarized our understanding of the unique properties and regulated activity of ISCs. A plethora of genetic studies have provided instrumental insights into the signalling networks that govern intestinal homeostasis. Crypt cells display substantial plasticity that is influenced by signals from the stem cell niche. Perturbations within these signalling pathways, most prominently the Wnt cascade, can induce tumourigenesis. A better understanding of the relationships and interconnectivity between homeostatic signalling and distinct aspects of tumour initiation and progression will be critical in the discovery of potential targets and development of strategies for therapeutic intervention.

## Data accessibility

This article has no additional data.
